# Protective Effects of Carbon Dots Derived from Phellodendri Chinensis Cortex Carbonisata against *Deinagkistrodon acutus* Venom-Induced Acute Kidney Injury

**DOI:** 10.1186/s11671-019-3198-1

**Published:** 2019-12-16

**Authors:** Meiling Zhang, Jinjun Cheng, Ziwei Sun, Hui Kong, Yue Zhang, Suna Wang, Xiaoke Wang, Yan Zhao, Huihua Qu

**Affiliations:** 1 School of Traditional Chinese Medicine, Beijing University of Chinese Medicine, Beijing, 100029 China; 2 School of Life Sciences, Beijing University of Chinese Medicine, Beijing, 100029 China; 3 Center of Scientific Experiment, Beijing University of Chinese Medicine, Beijing, 100029 China

**Keywords:** Acute kidney injury, Phellodendri Chinensis Cortex Carbonisata-carbon dots, Protective effect, *Deinagkistrodon acutus* venom

## Abstract

**Background:**

As an emerging nanomaterial, carbon dots (CDs) have been the focus of tremendous attention for biomedical applications. However, little information is available on their bioactivity of inhibiting acute kidney injury (AKI) induced by snake venom.

**Methods:**

This study reports the development of a green, one-step pyrolysis process to synthesize CDs using Phellodendri Chinensis Cortex (PCC) as the sole precursor, and their potential application as a protectant against *Deinagkistrodon acutus (D. acutus)* venom-induced AKI was investigated for the first time. The AKI model was established by injecting *D. acutus* venom into the abdominal cavity of mice and the potential protective effects of PCC Carbonisata-CDs (PCCC-CDs) on renal abnormalities including dysfunction, inflammatory reactions, tissue damage, and thrombocytopenia at six time points (1, 3, and 12 h, and 1, 2, and 5 days) were investigated.

**Results:**

These results demonstrated that PCCC-CDs significantly inhibited the kidney dysfunction (reduced serum creatinine (SCR), blood urea nitrogen (BUN), urinary total protein (UTP), and microalbuminuria (MALB) concentrations) and the production of chemoattractant (monocyte chemotactic protein 1 (MCP-1)), proinflammatory cytokines (interleukin (IL)-1β), and anti-inflammatory cytokine (IL-10) in response to intraperitoneal injection of *D. acutus* venom. The beneficial effect of PCCC-CDs on the envenomed mice was similar to that on the change in renal histology and thrombocytopenia.

**Conclusions:**

These results demonstrated the remarkable protective effects of PCCC-CDs against AKI induced by *D. acutus* venom, which would not only broaden the biomedical applications of CDs but also provide a potential target for the development of new therapeutic drugs for AKI induced by *D. acutus* snakebite envenomation.

## Introduction

*Deinagkistrodon acutus (D. acutus)*, belonging to the family Viperidae, is considered one of the most dangerous terrestrial snakes in China [1]. Envenomation by *D. acutus* is associated with a series of symptoms such as haemorrhage, thrombocytopaenia, and possible direct damage to the kidney [2, 3]. Acute kidney injury (AKI) is the most serious systemic effect and common complication of envenomation by *D. acutus* that directly leads to persistent kidney dysfunction and high morbidity [4, 5]. The current topical treatment involves the use of horse-derived hyperimmune antivenin as an antidote. However, its effectiveness is limited in neutralizing local tissue damage, especially in the occurrence of AKI, and has several unsatisfactory effects such as anaphylactic and pyrogenic reactions [6]. In addition, the relatively high cost and poor stability of antivenin also contribute to the unsatisfactory treatment of people bitten by *D. acutus*, especially in the wild or rural areas [7–9]. Therefore, there is an urgent need for effective, safe, and affordable complementary medicines for the treatment of *D. acutus* venom-induced AKI.

Carbon dots (CDs), a new class of carbon nanomaterials with a size < 10 nm, were serendipitously discovered by separation and purification of single-walled carbon nanotubes in 2004 [10] and have attracted much interest over the last decade because of their remarkable novel properties such as appreciable biocompatibility, low toxicity, high water solubility, and abundant raw materials [11–13]. The advent of CDs has contributed to research on the development of various “smart” nanosystems mainly including those for bioimaging [14], biomedicine [15], drug delivery [16], and photocatalysis [17].

Of note, the development of CDs with inherent bioactivity potential provides many strategies for the discovery of a new generation of drugs for the effective control or treatment of some diseases because of the remarkable aforementioned advantages. In several bioactivities such as antibacterial to treat bacterial keratitis [18], haemostatic [19], peroxidase-like [20], anticancer, antiviral, and anti-inflammatory activities [21] have been reported. These effects have attracted the attention of scientists to study additional pharmaceutical and biomedical applications of CDs. Especially, the alleviating activities of CDs derived from Schizonepetae Spica Carbonisata [22] on *D. acutus* venom-induced haemorrhage have provided a new perspective on the investigation of the beneficial effects of CDs on AKI induced by *D. acutus* snakebite, which remained less understood until now.

Phellodendri Chinensis Cortex (PCC) Carbonisata (PCCC)-CDs is synthesized by direct pyrolysis of PCC (a type of traditional Chinese medicine, which has been used for > 1000 years) using a one-step pyrolysis treatment. PCCC-CDs are hypotoxic CDs ranging in diameter from 1.2 to 4.8 nm. PCCC-CDs have been reported to possess remarkable haemostatic effects, which has not only broadened the biomedical application of CDs but also pioneered the elucidation of the haemostatic material basis of PCCC [23]. Of note, PCCC, a traditional Chinese medicine prepared by charcoal processing, was first recorded in the *Taiping Holy Prescriptions for Universal Relief* (978–992 AD, in China). The safety profile and satisfactory therapeutic efficacy of PCCC, such as haemostasis and anti-inflammation, encouraged its continued clinical application for more than 1000 years, which was acknowledged in the *Pharmacopoeia of the People’s Republic of China* (PPRC, 2015). However, the study of additional underlying bioactivities of PCCC-CDs has been a challenge. Especially, there is little information on the inhibition of AKI induced by *D. acutus* envenomation.

In addition, snakebite envenomation has been reported to possibly jeopardize renal physiology directly through the nephrotoxic components or by activation or modulation of immune and inflammatory mediators [24]. These effects are mainly on biomedical parameters [25] (serum creatinine (SCR), blood urea nitrogen (BUN), urinary total protein (UTP), and microalbuminuria (MALB) concentration), inflammatory cytokines (interleukin (IL)-1β), anti-inflammatory cytokine (IL-10), and monocyte chemotactic protein 1 (MCP-1), the change in renal histology, and platelet (PLT) count. In the present study, we synthesized PCCC-CDs using a green, one-step pyrolysis method and, for the first time, investigated their protective effects against the development of these abnormalities in AKI induced by intraperitoneal injection of mice with *D. acutus* venom.

## Material and Methods

### Chemicals

The PCC material was purchased from the Beijing Qiancao Herbal Pieces Co., Ltd. (Beijing, China), and the PCCC was prepared in our laboratory. Dialysis membranes with a molecular weight of 1000 Da were purchased from Beijing Ruida Henghui Technology Development Co., Ltd. (Beijing, China). The cell counting kit (CCK)-8 was purchased from Dojindo Molecular Technologies, Inc. (Kumamoto, Japan). The lyophilized snake venom of *D. acutus* was provided by An Ren Snake Farm (Yujiang County, Yingtan, Jiangxi, China) for experimental use. Other analytical grade chemical reagents were obtained from Sinopharm Chemical Reagents Beijing (Beijing, China). Mouse MCP-1, IL-10, and IL-1β enzyme-linked immunosorbent assay (ELISA) kits were purchased from Cloud-Clone Crop. (Wuhan, China). All the experiments were performed using deionized water (DW).

### Animals

Animal management and research protocols were supported and approved by the Institutional Committee of Ethics of Animal Experimentation of the Beijing University of Chinese Medicine. Male Kunming mice (weighing 30.0 ± 2.0 g) were purchased from Beijing Vital River Laboratory Animal Technology Co., Ltd., with a laboratory animal certificate of conformity, and were kept in cages at constant temperature and humidity on a 12-h light/dark cycle with *ad libitum* access to food and water.

### Preparation of Venom Solution

Lyophilized venom was dissolved in normal saline with mild mixing for 20 min (final concentration: 5 mg/mL) and stored at −20 °C until needed.

### Preparation of PCCC-CDs

**Fig. 1:**
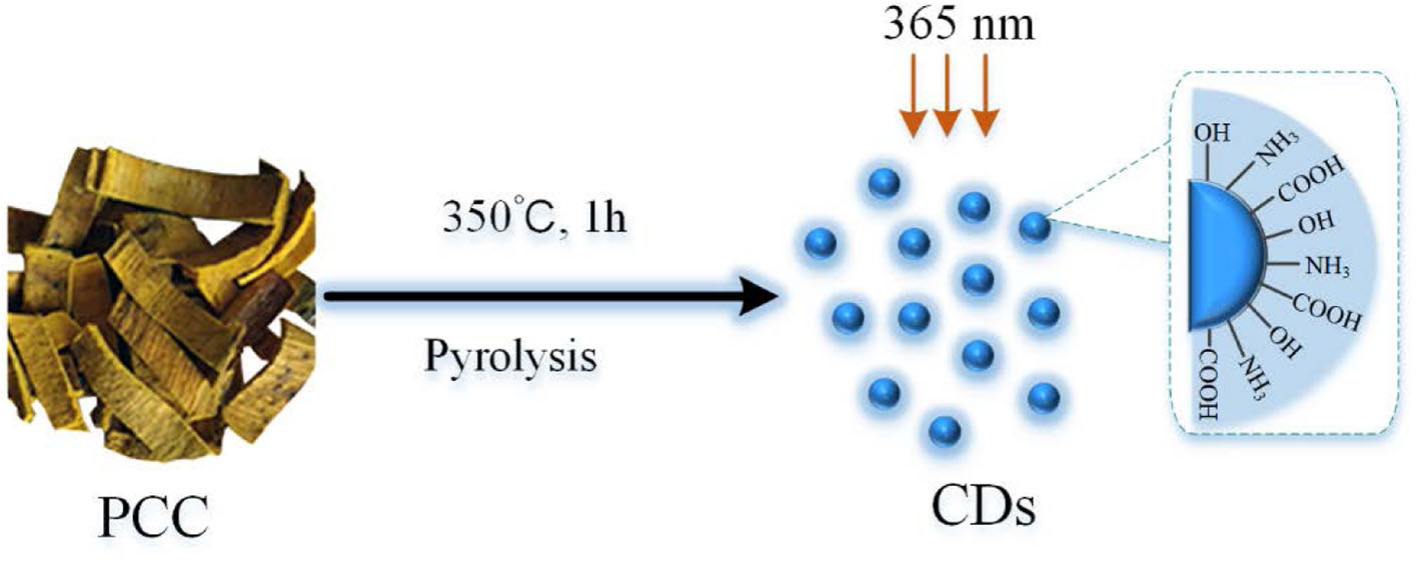
Illustration of the formation process of carbon dots (CDs) from Phellodendri Chinensis Cortex (PCC) by pyrolysis treatment

The PCCC was prepared using a one-step pyrolysis method with PCC as the sole precursor according to previous methods [23]. Briefly, the PCC samples were placed in separate porcelain crucibles and heated for 1 h at 350 °C in a pre-heated furnace. After cooling to 30 °C, the obtained homogenous black residues were ground into fine powder and boiled twice in water both at 100 °C for 1 h per time. Then, the solution was collected by pre-filtering the suspension through a 0.22 μm cellulose acetate membrane. Non-carbonaceous impurities were removed by dialysis against DW for 72 h (retained molecular weight: 1000 Da). The PCCC-CDs solution was concentrated and stored at 4 °C until use. The flow diagram in Fig. 1 illustrates the above process.

### Characterization of PCCC-CDs

The morphology of the CDs was studied using a transmission electron microscope (TEM, JEM-2100 electron, JEOL, Japan) with electron energy of 200 kV. Structural details were examined using high-resolution TEM (HRTEM) using a Tecnai G2 20 electron microscope (FEI, USA). The ultraviolet-visible (UV-Vis) spectrum and fluorescence properties were recorded and measured using UV-Vis (CECIL, Cambridge, UK) and fluorescence (F-4500, Tokyo, Japan) spectroscopy, respectively. Fourier transform infrared (FTIR) spectroscopy was used to analyse the surface functional groups information at a spectral window between 400 and 4000 cm^− 1^.

### Cytotoxicity Assays

Human L02 hepatocyte and the human embryonic kidney 293 T cell lines were used to assess the potential cytotoxicity of PCCC-CDs using a CCK-8 assay. L02 cells were cultured in Roswell Park Memorial Institute 1640 (RPMI 1640) medium containing 10% foetal bovine serum (FBS) while 293 T cells were grown in Dulbecco’s modified Eagle’s medium (DMEM) with high glucose, containing 10% FBS. Both cell lines were incubated at 37 °C in humidified 5% CO_2_.

L02 and 293 T cells were seeded at a density of 2.0 × 10^4^ cells/well in 96-well plates and cultured at 37 °C in a humidified atmosphere of 5% CO_2_ for 24 h. Then both cell types were treated with 100 μL of different concentrations (5000, 2500, 1250, 625, 156, 78, 39, and 19.5 μg/mL) of the PCCC-CD solutions in serum-free medium and were incubated for another 24 h. Subsequently, the medium containing the PCCC-CDs was removed and the cells were washed with phosphate-buffered saline (PBS) twice. Cytotoxicity was determined by reading the plate at 450 nm immediately after adding 10 μL of the CCK-8 reagent and incubating for 4 h at 37 °C.

### *D. acutus* Venom-Induced AKI and Treatment

#### *D. acutus* Venom-Induced AKI Model

The AKI mouse model was established by intraperitoneally injecting mice with *D. acutus* snake venom. The mouses were randomly divided into the following five groups of 36 animals each: control; model; and high-, medium-, and low-dose PCCC-CDs treatment groups. The model group received *D. acutus* venom at 1 mg/kg (0.15 mg/mL, 0.2 mL) body weight and 0.5 mL normal saline intraperitoneally, while the high-, medium-, and low-dose PCCC-CD treatment groups received an equivalent volume of snake venom and PCCC-CD extract at doses of 8.0, 4.0, and 2.0 mg/kg, respectively. Mice injected intraperitoneally with only normal saline (NS) served as the controls. Six mice from each group were sacrificed 1, 3, and 12 h after administration of NS, *D. acutus* venom, and PCCC-CDs on the schedule described above, while mouse sacrificed on day 1, 2, and 5 were continuously administered with NS, *D. acutus* snake venom, and PCCC-CDs twice a day.

### Analysis of UTP and MALB Concentrations

After administration of the respective treatments, animals in the control, model, and PCCC-CDs- (4.0 mg/kg) treated groups were immediately placed in appropriate metabolic cages for urine collection until the incubation period ended (24 h). The concentrations of UTP and MALB were analysed using an automatic biochemical analyser.

### Analysis of Biomarkers of Kidney Function

Kidney function was assessed by measuring the levels of SCR and BUN. Before the mice were euthanized, blood samples were withdrawn from the retro-orbital plexus and then placed into plastic tubes for at least 4 h at 4 °C. Serum was obtained by centrifugation at 750×*g* for 15 min, and BUN and SCR levels were analysed using an automatic biochemical analyser.

### Detection of Inflammatory Cytokine Levels

The right kidneys of the mice were removed, rapidly frozen in dry ice and stored at −80 °C until use in the following procedures. Tissue samples (100 mg) from the different groups were homogenized with PBS on ice and then centrifuged at 750×*g* for 15 min. The supernatants were collected to determine levels of MCP-1, IL-10, and IL-1β using respective ELISA kits according to the manufacturer’s instructions.

### Kidney Histology

The mouse left kidney tissue samples were fixed in 10% neutral-buffered formalin at 4 °C for more than 48 h, dehydrated, embedded in paraffin, cut into sections, and then stained with haematoxylin and eosin (H&E). Morphological changes were compared among the control, model, and the PCCC-CDs-treated groups.

### Thrombocytopenic Activity

The PLT was performed on blood withdrawn from the mice from the retro-orbital plexus and detected using an automated haematology analyser (XS-800i, Sysmex Corporation Co., Ltd., Kobe, Japan).

### Statistical Analysis

Statistical analysis was performed using the Statistical Package for the Social Sciences (SPSS, version 13.0). The normally distributed data and homogeneous variances are expressed as means ± standard deviation (SD). A one-way analysis of variance (ANOVA) followed by the least significant difference (LSD) test was used for multiple comparisons. *P* < 0.05 was considered statistically significant.

## Results

### Characterization of PCCC-CDs

**Fig. 2:**
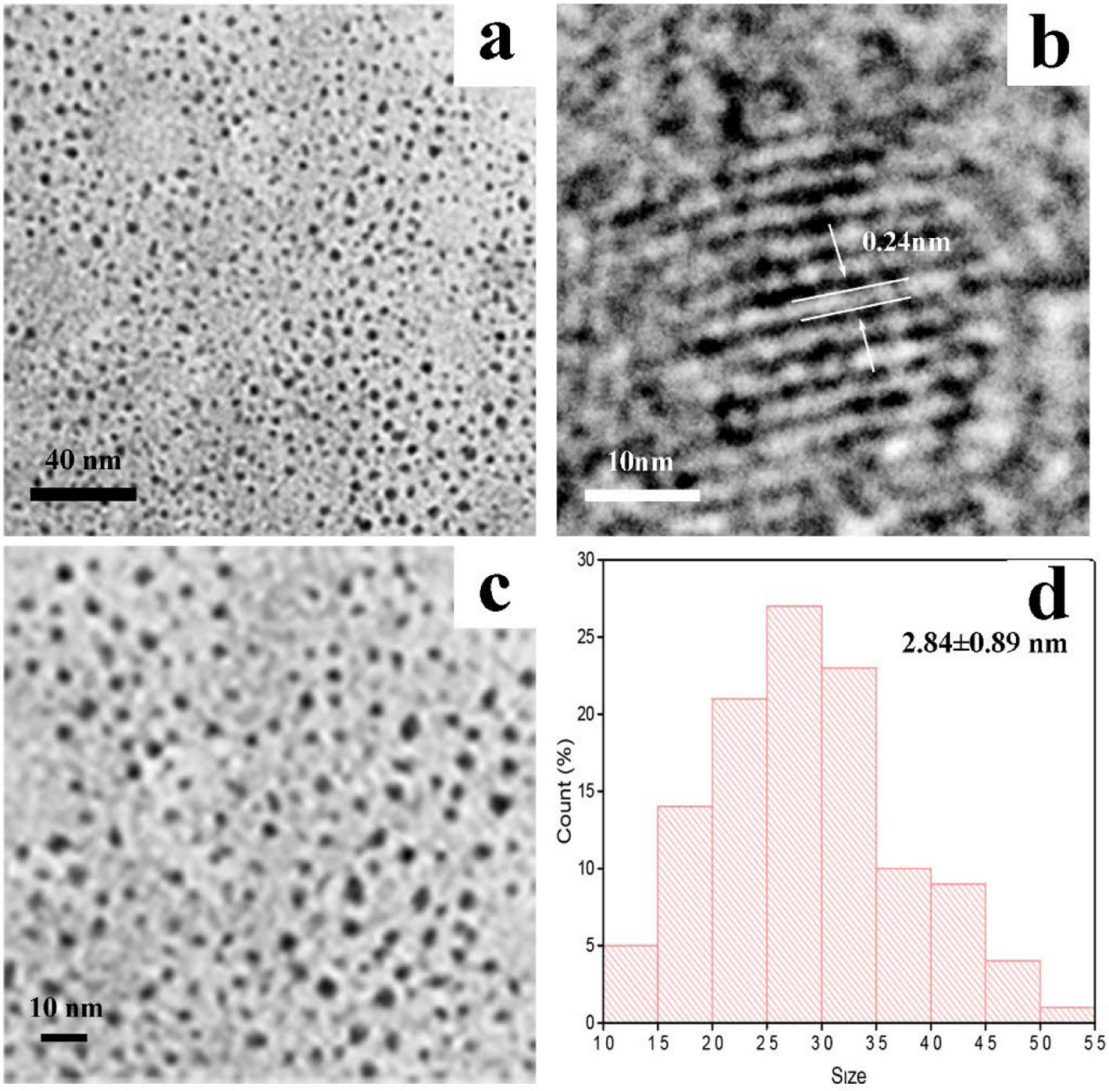
**a** and **c** Transmission electron microscopy (TEM) images of Phellodendri Cortex Carbonisatus carbon dots (PCCC-CDs), **b** High-resolution TEM image of PCCC-CDs, **d** Size distribution of PCCC-CDs

TEM was used to directly observe the morphology and size distribution of PCCC-CDs (Fig. 2a, c, and d). The CDs prepared were spherical and uniform in size, with most at 2.84 ± 0.89 nm without distinct aggregations. The HRTEM image showed lattice fringes (0.24 nm) of the CDs, which corresponded to graphitic carbon as shown in Fig. 2b.

**Fig. 3:**
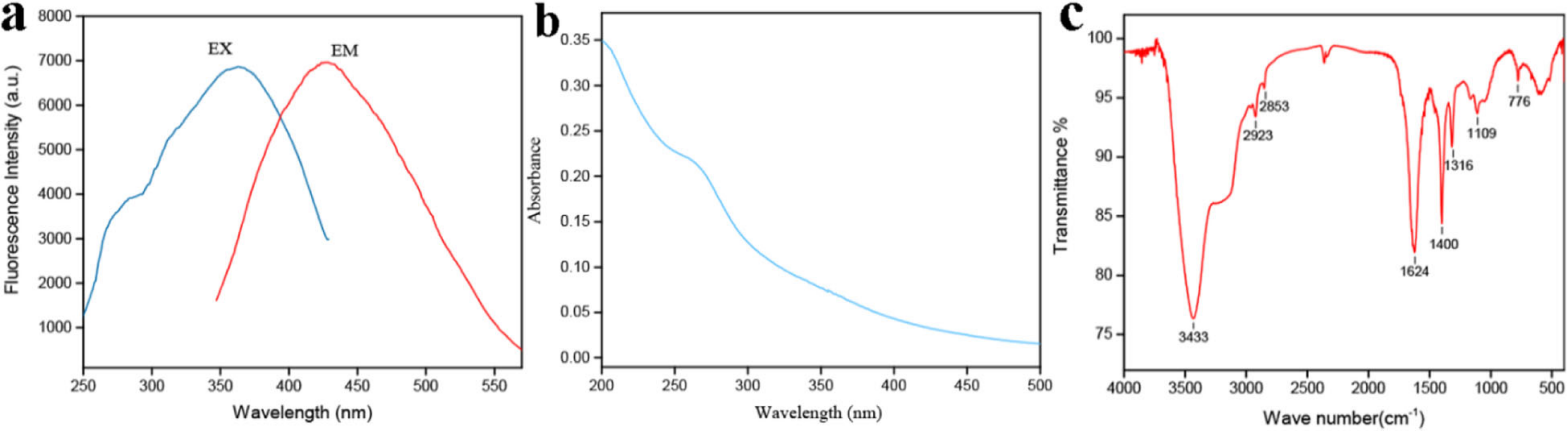
Optical properties of Phellodendri Cortex Carbonisatus carbon dots (PCCC-CDs). **a** Fluorescence spectra, **b** ultraviolet-visible spectra (UV-Vis) and **c** Fourier transform infrared spectrum (FTIR)

As depicted in Fig. 3a, the CDs exhibited distinctly blue fluorescence with a peak maximum at 445 nm following 370 nm excitation. Accordingly, the optical information on the PCCC-CDs displayed in the UV-Vis spectrum showed a strong absorption peak at 265 nm corresponding to π-π* transition of conjugated organic molecules on the CDs surfaces (Fig. 3b).

In addition, the functional groups of the formulated CDs were identified based on the FTIR spectrum in detail, as depicted in Fig. 3c. The peak at 3433 cm^− 1^ was assigned to the absorption bands of O–H and N–H stretching vibrations while C–H stretching vibrations appeared at 2923 cm^− 1^ and 2853 cm^− 1^. In addition, the absorption peak at 1624 cm^− 1^ indicated the presence of C=O. C–O–C bonds were observed at 1109 cm^− 1^, and the peak at 1400 cm^− 1^ was attributed to C–N stretching. These findings were consistent with those of previous reports.

### In Vitro Cytotoxicity

**Fig. 4:**
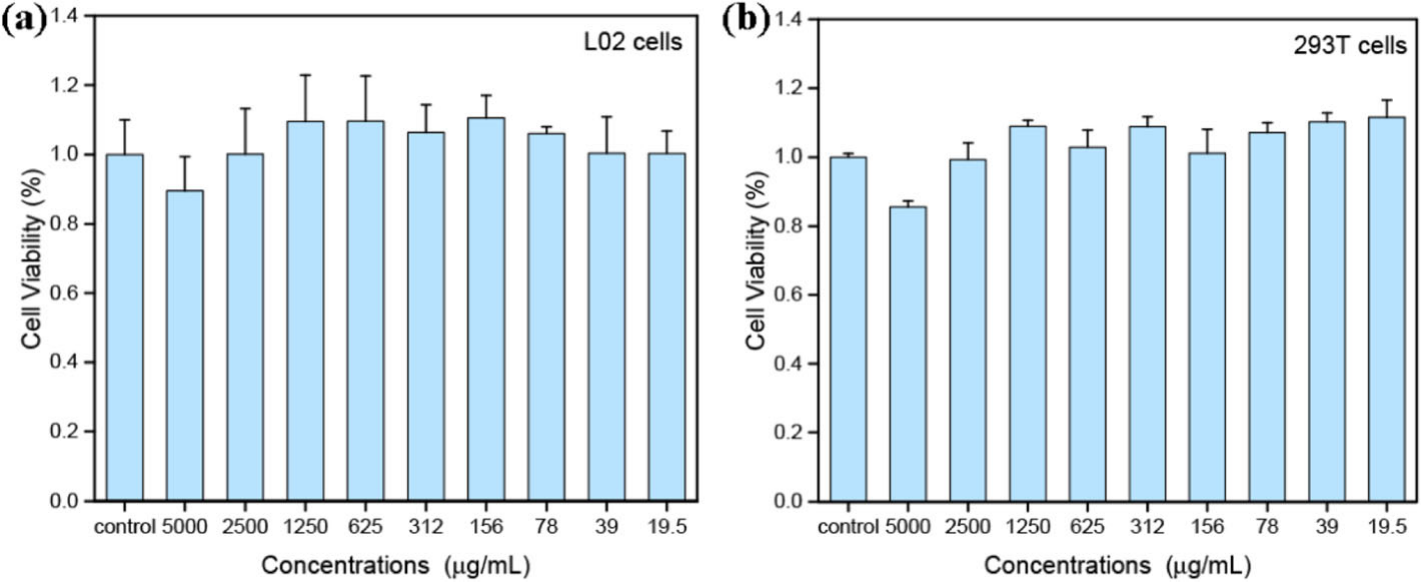
Cell viability by CCK-8 assay of (**a**) L02 cells and (**b**) 293 T cells incubated with PCCC-CDs for 4 h

To assess the cytotoxicity, L02 and 293 T cells were exposed to various concentrations of the PCCC-CDs for 24 h and viability was examined using the CCK-8 assay. As illustrated in Fig. 4a, the viability of L02 cells treated with PCCC-CDs was > 85% compared with that of the control cells, even at a concentration as high as 5 mg/mL. Similarly, the PCCC-CDs did not affect 293 T cell growth at concentrations of up to approximately 2500 μg/mL (Fig. 4b). These results indicated that the PCCC-CDs showed low cytotoxicity.

### Effect of PCCC-CDs on UTP and MALB Concentrations

**Fig. 5:**
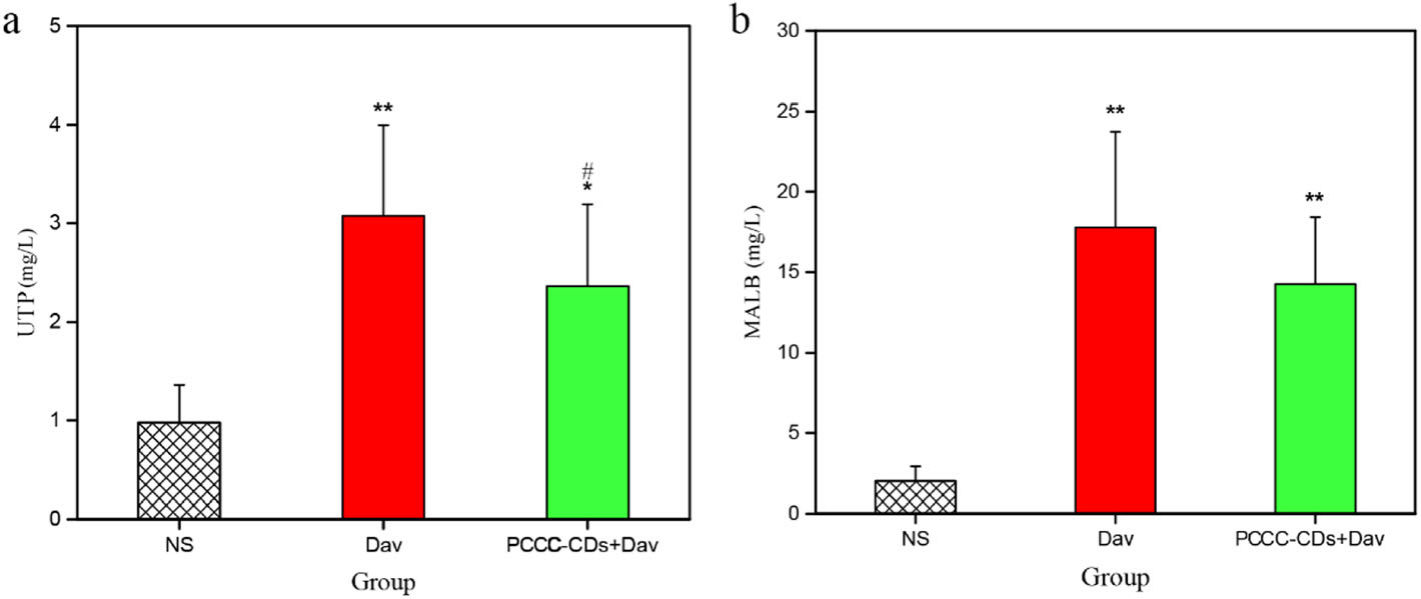
Effects of Phellodendri Chinensis Cortex Carbonisata-carbon dots (PCCC-CDs) on urinary total protein (UTP) and microalbuminuria (MALB) in the urine. **a** UTP and **b** MALB. Mice were treated with normal saline (NS), *D.acutus* venom (Dav, 1 mg/kg) and PCCC-CDs (4 mg/kg) + Dav (1 mg/kg). Data are represented as mean ± SD of six animals of each group. *p < 0.05 and ** *p <* 0.01 compared with control group treated with NS. ^#^*p <* 0.05 compared with Dav group

Compared with the UTP level in the control group (0.98 ± 0.38 mg/L), UTP levels significantly increased in the *D. acutus* venom-treated (3.08 ± 0.92 mg/L, *P* < 0.01) and PCCC-CDs (2.36 ± 0.83 mg/L, *P* < 0.05) groups (Fig. 5a). Furthermore, the UTP level decreased after PCCC-CD treatment compared with the levels following venom administration (*P* < 0.05). In addition, the level of MALB obviously increased 24 h after intraperitoneal injection of *D. acutus* venom (17.78 ± 5.96 mg/L, *P* < 0.01) compared to that of the control group (2.02 ± 0.91 mg/L). In contrast, the PCCC-CD-treated mice showed a decrease tendency in the level of MALB (14.25 ± 4.16 mg/L, Fig. 5b).

### PCCC-CDs Relieved Kidney Dysfunction in *D. acutus* Venom-Induced AKI Mice

**Fig. 6:**
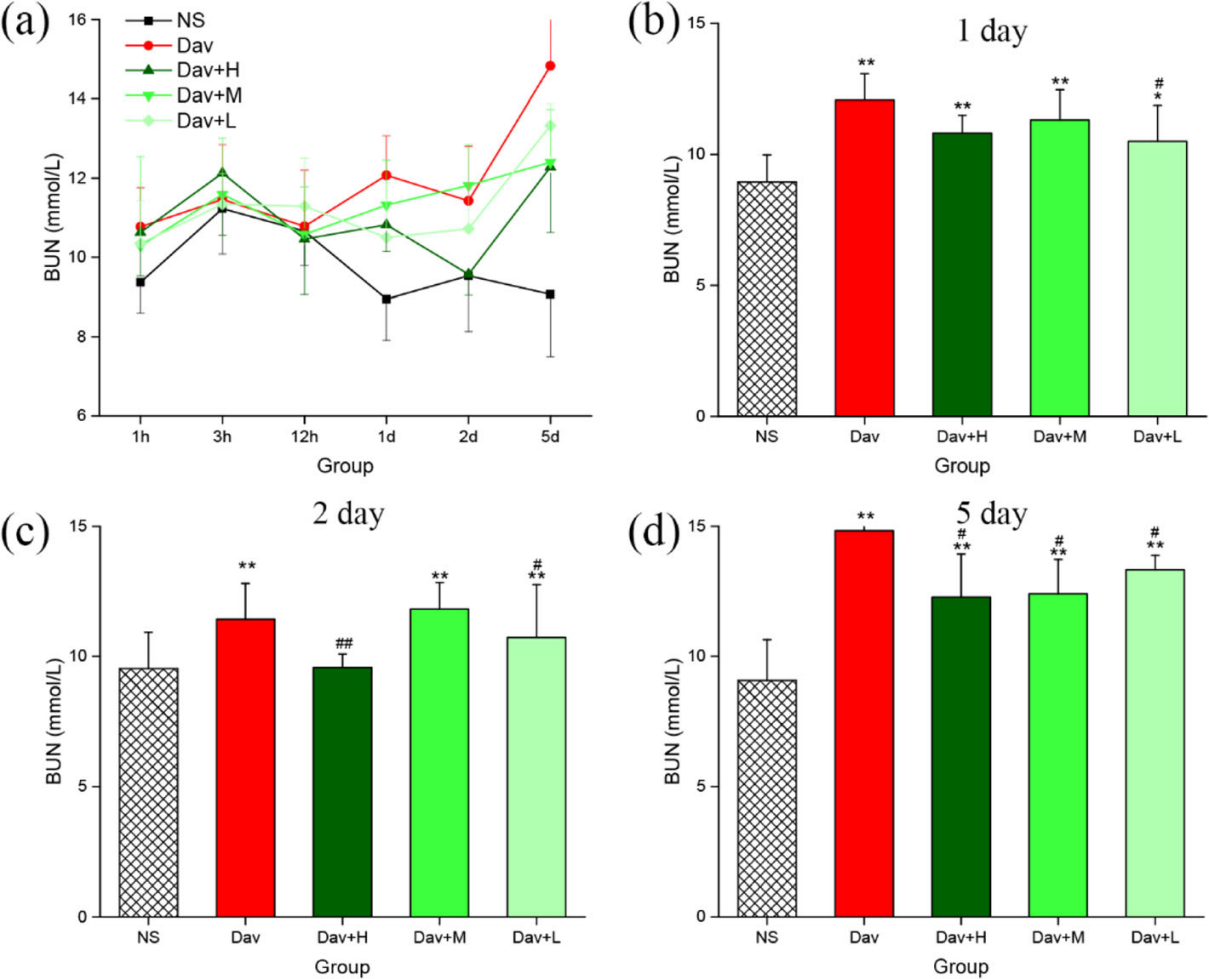
Effects of Phellodendri Chinensis Cortex Carbonisata - carbon dots (PCCC-CDs) on blood urea nitrogen (BUN). **a** BUN concentration changes in 1 h, 3 h, 12 h, 1 day, 2 day and 5 day. The levels of BUN in (**b**) 1 day (**c**) 2 day (**d**) 5 day. Mouse were treated with normal saline (NS), *D.acutus* venom (Dav, 1 mg/kg) and high- (H), medium- (M) and low (L) doses of PCCC-CDs + Dav (1 mg/kg). Data are represented as mean ± SD of six animals of each group. *p < 0.05 and ** *p <* 0.01 compared with control group treated with NS. ^#^*p <* 0.05 compared with Dav group

**Fig. 7:**
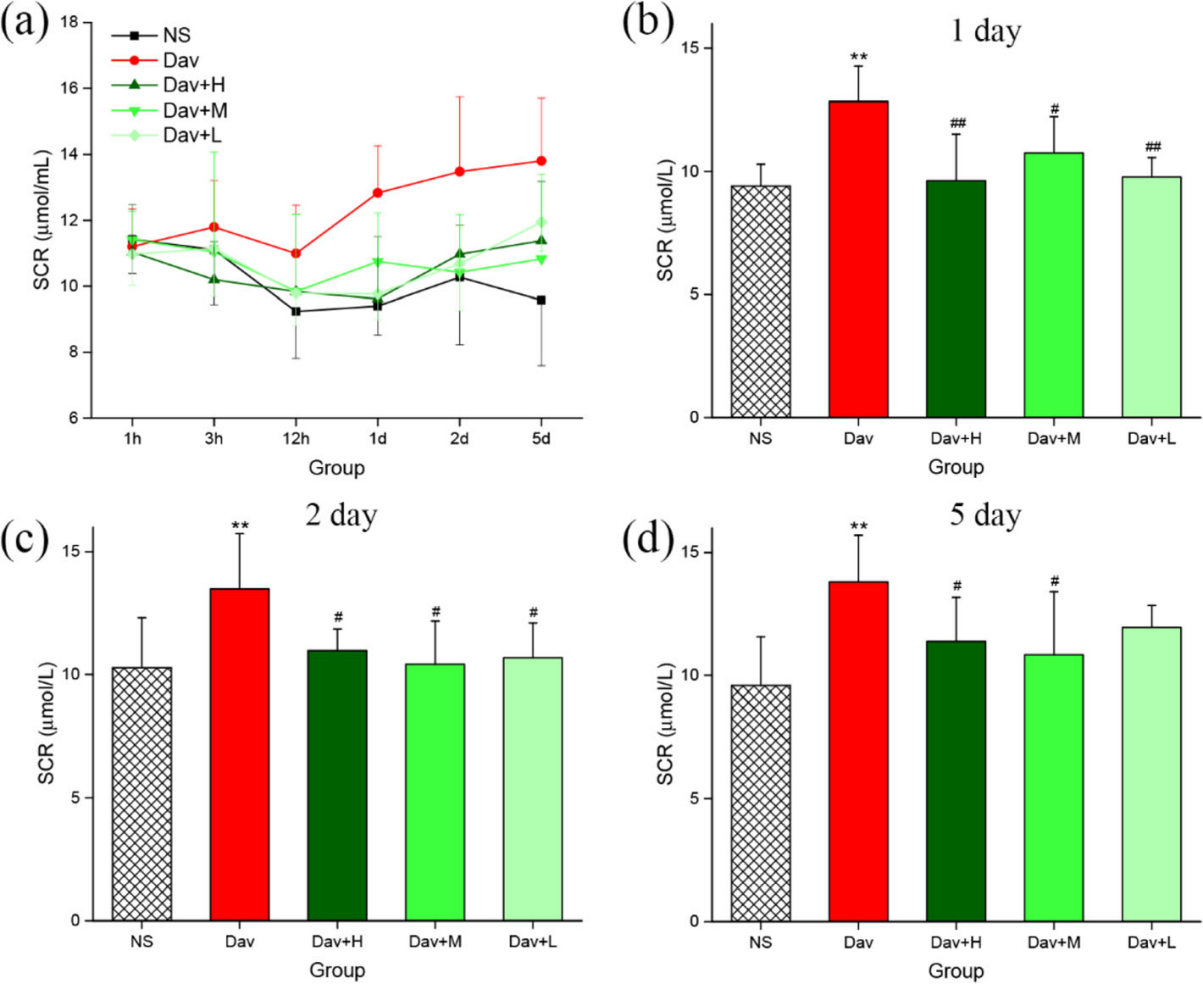
Effects of Phellodendri Chinensis Cortex Carbonisata-carbon dots (PCCC-CDs) on serum creatinine (SCR). **a** SCR concentration changes in 1 h, 3 h, 12 h, 1 day, 2 days, and 5 days. The levels of SCR in (**b**) 1 day (**c**) 2 days (**d**) 5 days. Mice were treated with normal saline (NS), *D.acutus* venom (Dav, 1 mg/kg) and high (H), medium (M), and low (L) doses of PCCC-CDs + Dav (1 mg/kg). Data are represented as mean ± SD of six animals of each group. *p < 0.05 and ** *p <* 0.01 compared with control group treated with normal saline (NS). ^#^*p <* 0.05 compared with Dav group

Renal function was assessed by measuring the levels of BUN and SCR, which are shown in Figs. 6 and 7. The mice treated with *D. acutus* venom had significantly higher levels of BUN (12.07 ± 1.00 mmol/L [1 day], *P* < 0.01; 11.43 ± 1.37 mmol/L [2 days], *P* < 0.01; 14.83 ± 2.53 mmol/L [5 days], *P* < 0.01) and SCR (12.83 ± 1.43 μmol/L [1 day], *P* < 0.01; 13.48 ± 2.26 μmol/L [2 days]; *P* < 0.01. 13.80 ± 1.90 μmol/L [5 days], *P* < 0.01) than the NS-treated mice did (BUN: 8.95 ± 1.04 [1 day], 9.53 ± 1.40 [2 days], and 9.07 ± 1.57 [5 days] mmol/L; SCR: 9.40 ± 0.89 [1 day], 10.27 ± 2.04 [2 days], and 9.85 ± 1.99 [5 days] μmol/L) from day 1 to 5 (Figs. 6a and 7a). Of note, compared with the model group, treatment with low-dose PCCC-CDs significantly inhibited the increase in levels of SCR (9.77 ± 0.79 μmol/mL, *P* < 0.01) and BUN (10.50 ± 1.38 mmol/L, *P* < 0.05), whereas high (9.62 ± 1.87 μmol/mL, *P* < 0.01) and medium (10.75 ± 1.48 μmol/mL, *P* < 0.05) doses inhibited the increase in SCR (Fig. 7b) but not BUN (Fig. 6b) induced by *D. acutus* venom on day 1. The levels of SCR (Fig. 7c) of the high-, medium-, and low-dose PCCC-CD-treated groups decreased (10.97 ± 0.88, 10.42 ± 1.75, and 10.68 ± 1.41 μmol/mL, respectively; *P* < 0.05) than in the model group in day 2. Furthermore, compared to the model group, BUN levels decreased significantly in the high- (9.57 ± 0.52 mmol/L, *P* < 0.01) and low-dose (10.72 ± 2.04 mmol/L, *P* < 0.05) PCCC-CDs group but not the medium-dose group (Fig. 6c) on day 2. As shown in Figs. 6d and 7d, inhibitory effects were observed on levels of both indexes at high (12.28 ± 1.65 mmol/L, *P* < 0.01 (BUN); 11.38 ± 1.80 μmol/mL, *P* < 0.05 (SCR), respectively) and medium (12.40 ± 1.33 mmol/L, *P* < 0.05 (BUN); 10.83 ± 2.57 μmol/mL, *P* < 0.05 (SCR), respectively) doses of PCCC-CDs. In addition, no significant difference was observed between the control and PCCC-CDs-treated groups in the SCR.

### PCCC-CDs Inhibited Cytokine Secretion

**Fig. 8:**
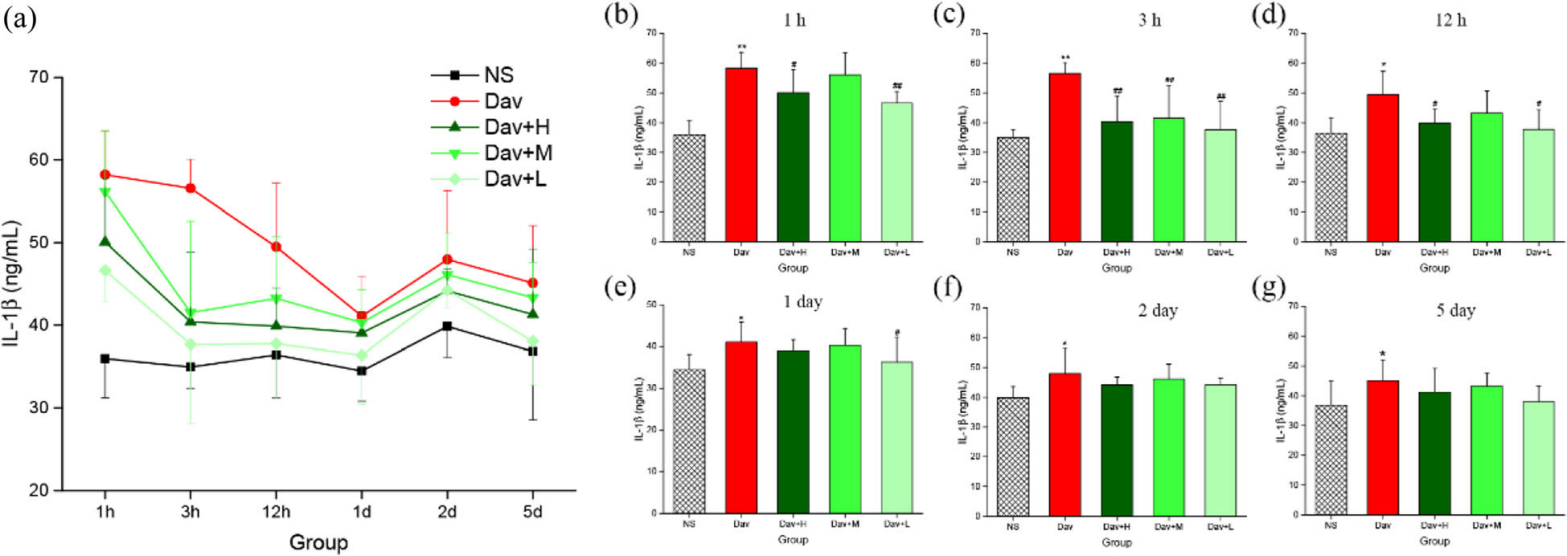
Effects of Phellodendri Chinensis Cortex Carbonisata-carbon dots (PCCC-CDs) on IL-1β level in the kidney tissue. **a** IL-1β level changes in 1 h, 3 h, 12 h, 1 day, 2 days, and 5 days. IL-1β levels in (**b**) 1 h, (**c**) 3 h, (**d**) 12 h, (**e**) 1 day, (**f**) 2 days, and (**g**) 5 days. Mice were treated with normal saline (NS), *D.acutus* venom (Dav, 1 mg/kg) and high (H), medium (M), and low (L) doses of PCCC-CDs + Dav (1 mg/kg). Data are represented as mean ± SD of six animals of each group. *p < 0.05 and ** *p* < 0.01 compared with control group treated with normal saline (NS). ^#^*p <* 0.05 compared with Dav group

The effects of three concentrations of PCCC-CDs on the production of chemoattractant (MCP-1) and proinflammatory (IL-1β) and anti-inflammatory (IL-10) cytokines in response to injections of *D. acutus* venom were investigated. Figure 8 showed that the injection of venom increased the IL-1β released in the mouse model (1 h: 58.19 ± 5.35 ng/mL, *P* < 0.01; 3 h: 56.57 ± 3.54 ng/mL, *P* < 0.01; 12 h: 49.48 ± 7.74 ng/mL, *P* < 0.05; day 1: 41.09 ± 4.82 ng/mL, *P* < 0.05; day 2: 47.96 ± 8.33 ng/mL, *P* < 0.05; day 5: 45.11 ± 6.95 ng/mL, *P* < 0.05) compared with levels in the NS-treated mouse (1 h: 35.96 ± 4.72 ng/mL, 3 h: 34.94 ± 2.58 ng/mL, 12 h: 36.42 ± 5.25 ng/mL, day 1: 34.47 ± 3.67 ng/mL, day 2: 39.84 ± 3.71 ng/mL, day 5: 36.82 ± 8.27 ng/mL). As shown in Fig. 8b–d, comparison with the *D. acutus* venom-induced group showed that 1-,3-, and 12-h exposure to high- (50.09 ± 7.68 ng/mL, *P* < 0.05 [1 h]; 40.36 ± 8.51 ng/mL, *P* < 0.01 [3 h]; 39.87 ± 4.64 ng/mL, *P* < 0.05 [12 h], respectively) and low- (46.64 ± 3.83 ng/mL, *P* < 0.01 [1 h]; 37.65 ± 9.61 ng/mL, *P* < 0.01 [3 h]; 38.75 ± 6.64 ng/mL, *P* < 0.05 [12 h], respectively) dose PCCC-CDs significantly decrease the levels of IL-1β. In addition, medium-dose PCCC-CDs significantly reduced the level of IL-1β (41.50 ± 11.08 ng/mL, *P* < 0.01) compared with that of venom-treated mice at 3 h but not at other time points.

**Fig. 9:**
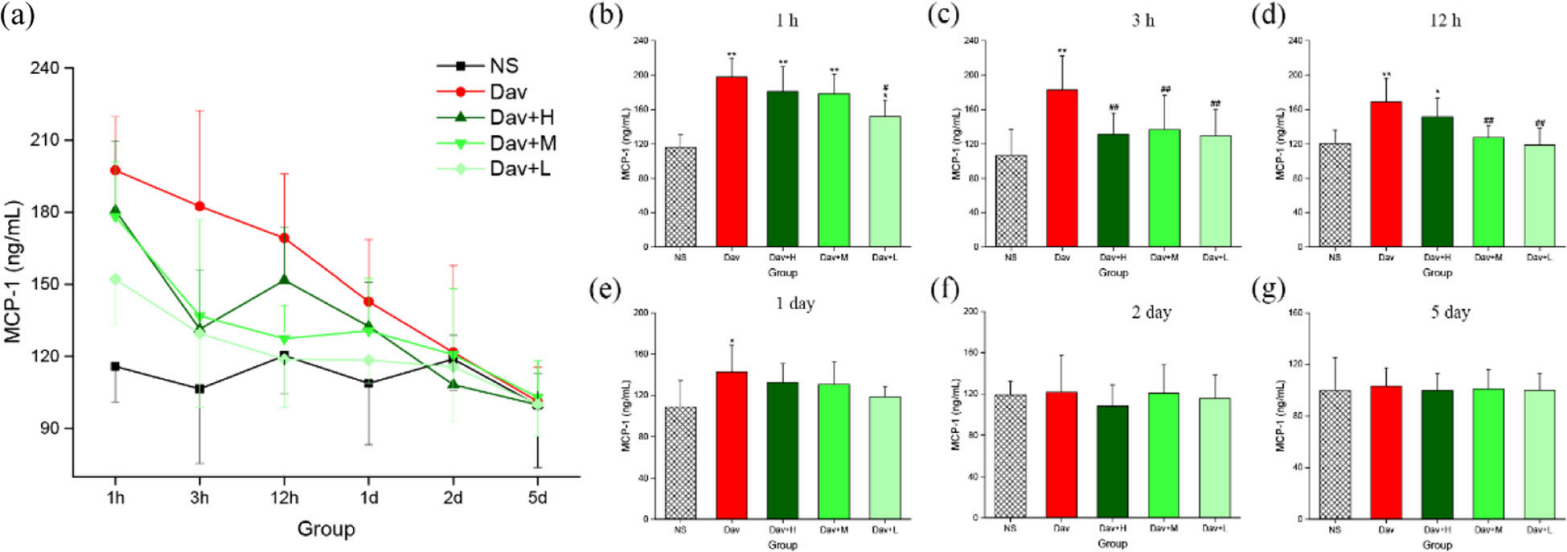
Effects of Phellodendri Chinensis Cortex Carbonisata-carbon dots (PCCC-CDs) on the level of IL-10 in the kidney tissue. **a** IL-10 level changes in 1 h, 3 h, 12 h, 1 day, 2 days, and 5 days. The level of IL-10 in (**b**) 1 h, (**c**) 3 h, (**d**) 12 h, (**e**) 1 day, (**f**) 2 days and (**g**) 5 days. Mice were treated with normal saline (NS), *D.acutus* venom (Dav, 1 mg/kg) and high (H), medium (M), and low (L) doses of PCCC-CDs + Dav (1 mg/kg). Data are represented as mean ± SD of six animals of each group. *p < 0.05 and ** *p* < 0.01 compared with control group treated with normal saline (NS). ^#^*p <* 0.05 compared with Dav group

The levels of the anti-inflammatory cytokine IL-10 dramatically increased in the *D. acutus* venom-treated group at different time points (23.27 ± 0.72 ng/mL, *P* < 0.01; 22.03 ± 0.96 ng/mL, *P* < 0.05; 21.76 ± 1.99 ng/mL, *P* < 0.05; 26.31 ± 6.55 ng/mL, *P* < 0.01; 3 h, 12 h, day 1, and day 2, respectively) compared with NS-treated group (Fig. 9). In sharp contrast, treatment with high- and medium-dose PCCC-CDs (17.17 ± 4.04 and 17.25 ± 5.64 ng/mL, respectively, both *P* < 0.05) inhibited venom-induced secretion of IL-10 at 3 h while low-dose PCCC-CDs inhibited the levels at 3 h, 12 h, and day 1 (17.17 ± 5.24, 17.83 ± 4.11, and 18.31 ± 2.14 ng/mL, respectively, all *P* < 0.05). No significant difference was observed between the PCCC-CDs-treated and NS group.

**Fig. 10:**
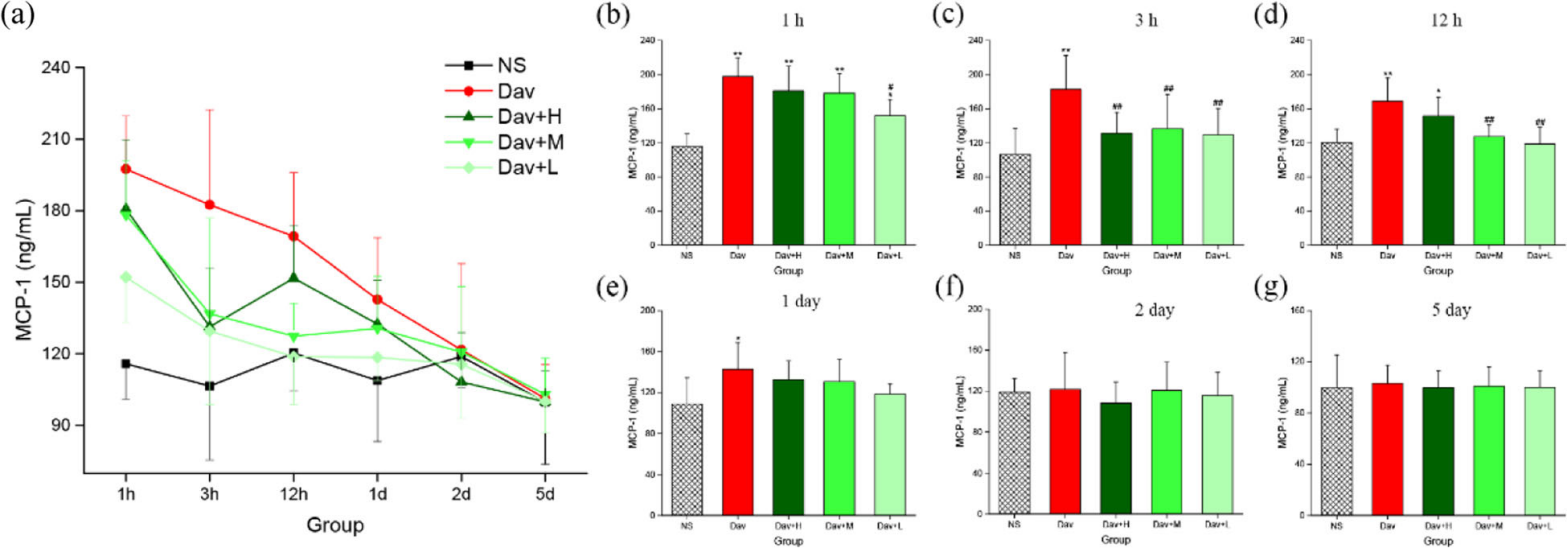
Effects of Phellodendri Chinensis Cortex Carbonisata-carbon dots (PCCC-CDs) on MCP-1 level in the kidney tissue. **a** MCP-1 level changes in 1 h, 3 h, 12 h, 1 day, 2 days, and 5 days. MCP-1 levels in (**b**) 1 h, (**c**) 3 h, (**d**) 12 h, (**e**) 1 day, (**f**) 2 days, and (**g**) 5 days. Mice were treated with normal saline (NS), *D.acutus* venom (Dav, 1 mg/kg) and high (H), medium (M), and low (L) doses of PCCC-CDs + Dav (1 mg/kg). Data are represented as mean ± SD of six animals of each group. *p < 0.05 and ** *p* < 0.01 compared with control group treated with normal saline (NS). ^#^*p <* 0.05 compared with Dav group

In addition, Fig. 10 shows that injection of *D. acutus* venom significantly increased MCP-1 release in the model group (1 h: 197.45 ± 22.34 ng/mL, *P* < 0.01; 3 h: 182.42 ± 12.94 ng/mL, *P* < 0.01; 12 h: 169.20 ± 26.74 ng/mL, *P* < 0.01; 24 h: 142.81 ± 25.85 ng/mL, *P* < 0.05) compared with the NS-treated control (1 h: 115.82 ± 14.80 ng/mL; 3 h: 106.46 ± 13.76 ng/mL; 12 h: 120.35 ± 15.75 ng/mL; and 24 h: 108.81 ± 25.60 ng/mL). More remarkable, treatment of envenomed mice with the three PCCC-CD doses inhibited the increase in MCP-1 levels. The medium- (3 h: 136.84 ± 39.94 ng/mL, *P* < 0.01; 12 h: 127.48 ± 13.75 ng/mL, *P* < 0.01) and low- (1 h: 152.13 ± 18.89 ng/mL, *P* < 0.05; 3 h: 129.54 ± 30.85 ng/mL, *P* < 0.01; 12 h: 118.75 ± 19.96 ng/mL, *P* < 0.01) dose PCCC-CDs inhibited the increase in levels of MCP-1 at 1, 3, and 12 h, except for medium-dose, at 1 h (178.20 ± 22.79 ng/mL). Treatment with high-dose PCCC-CDs decreased effectively in the level of MCP-1 only at 3 h (131.42 ± 24.62 ng/mL, *P* < 0.01).

In contrast, IL-1β, IL-10, and MCP-1 levels of the PCCC-CDs-treated groups at certain time points were not significantly different from those of the model group, and showed a decreasing tendency.

### Effect of PCCC-CDs on the Inhibition of Thrombocytopenia

**Fig. 11:**
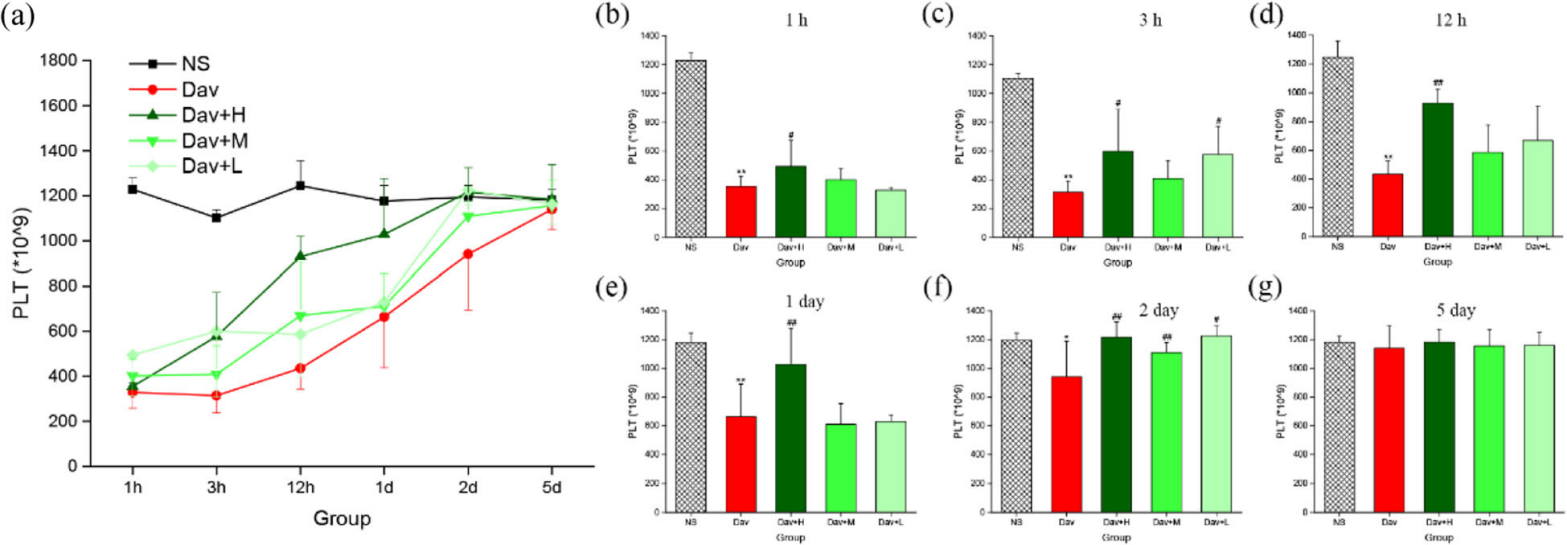
Effects of Phellodendri Chinensis Cortex Carbonisata-carbon dots (PCCC-CDs) on platelet (PLT) counts in the blood. **a** PLT changes in 1 h, 3 h, 12 h, 1 day, 2 days, and 5 days. PLT in (**b**) 1 h, (**c**) 3 h, (**d**) 12 h, (**e**) 1 day, (**f**) 2 days, and (**g**) 5 days. Mice were treated with normal saline (NS), *D.acutus* venom (Dav, 1 mg/kg) and high (H), medium (M), and low (L) doses of PCCC-CDs + Dav (1 mg/kg). Data are represented as mean ± SD of six animals of each group. *p < 0.05 and ** *p* < 0.01 compared with control group treated with normal saline (NS). ^#^*p <* 0.05 compared with Dav group

PLTs have a specific role in the pathogenesis of AKI; therefore, the PLT count was investigated and the results are shown in Fig. 11 [26]. Compared with the PLT values of the control group (1 h: [1228 ± 51] × 10^9^; 3 h: [1120 ± 36] × 10^9^; 12 h: [1245 ± 111] × 10^9^; day 1: [1177 ± 69] × 10^9^; day 2: [1195 ± 51] × 10^9^; and day 5: [1181 ± 46] × 10^9^), a drastic reduction occurred as early as 1 h after *D. acutus* venom administration, with a nadir occurring at 3 h. Subsequently, the PLT steadily increased up to day 5 (1 h: [354 ± 70] × 10^9^, *P* < 0.01; 3 h: [315 ± 77] × 10^9^, *P* < 0.01; 12 h: [435 ± 91] × 10^9^, *P* < 0.01; day 1: [663 ± 226] × 10^9^, *P* < 0.01; day 2: [941 ± 248] × 10^9^, *P* < 0.05; day 5: [1083 ± 89] × 10^9^). Of note, even at this time interval, the PLT values were significantly lower than those of control mice. In addition, administration of PCCC-CDs at a dose of 8 mg/kg markedly inhibited the venom-induced thrombocytopenia induced at 1 h ([435 ± 91] × 10^9^, *P* < 0.05), 3 h ([599 ± 290] × 10^9^, *P* < 0.05), 12 h ([929 ± 92] × 10^9^, *P* < 0.01), day 1 ([1028 ± 248] × 10^9^, *P* < 0.01), and day 2 ([1183 ± 89] × 10^9^, *P* < 0.01). This inhibitory effect was also observed at a dose of 2 mg/kg PCCC-CDs at 3 h and day 2 and 4 mg/kg PCCC-CDs at day 2, in addition to increased PLT. Although there was no significant difference between the model and medium-dose groups, an increasing tendency was observed at other different time points.

### Histopathological Observations

**Fig. 12:**
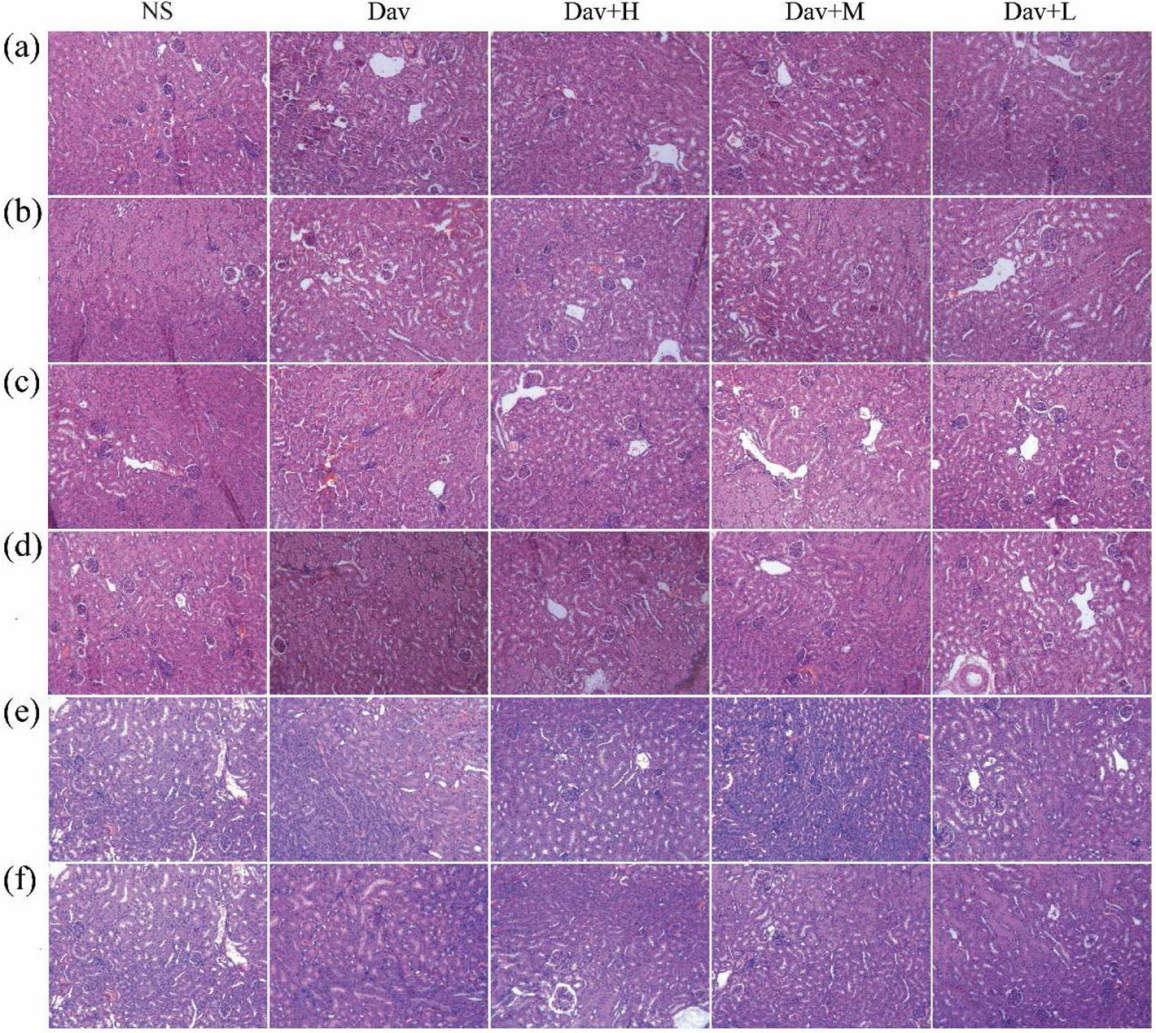
Effects of Phellodendri Chinensis Cortex Carbonisata-carbon dots (PCCC-CDs) on histopathological changes in kidney tissues in *D.acutus* venom (Dav)-induced AKI mice. After *D.acutus* venom challenged, kidney tissues from each experimental group were prepared for histological evaluation. Histological changes of kidney obtained from mice of different groups normal saline (NS), *D.acutus* venom (Dav, 1 mg/kg) and high (H), medium (M), and low (L) doses of PCCC-CDs + Dav (1 mg/kg) in (**a**) 1 h, (**b**) 3 h, (**c**) 12 h, (**d**) 1 day, (**e**) 2 days, and (**f**) 5 days

The renal injury in the venom group was histologically evaluated. As shown in Fig. 12a, in contrast to the NS-treated group, which showed normal glomeruli and tubular cellularity, marked changes were observed in the renal parenchyma of the *D. acutus* venom-treated group. These changes include marked haemorrhage, renal tubular dilation, and degeneration. Cotreatment with PCCC-CDs prevented *D. acutus* venom-induced renal damage, and the histopathological examination of the architecture of the renal tissues was almost normal with mild haemostasis, renal tubular dilation, and degeneration.

## Discussion

As an emerging nanomaterial, CDs are beginning to occupy an important niche as innovative materials for next-generation nanomedicines. Compared to traditional heavy-metal-based quantum dots, CDs are good candidates for biomedical application because of their unique characteristics that have considerable potential advantages in the development of novel medicines with relatively low toxicity [27, 28].

The derived PCCC-CDs particles were quasi-spherical and well-dispersed in water, with abundant functional groups present on the surface. This observation is consistent with previously reported findings [23]. In addition, the as-prepared CDs showed low toxicity against L02 and 237 T cells, which indicated its suitability for biomedical applications.

The current study is the first, to the best of our knowledge, to demonstrate the remarkable bioactivities of PCCC-CDs on AKI induced by *D. acutus* snakebite. *D. acutus* is widely called “five pacer” or “hundred pacer” in Chinese folk medicine on account of the folkloric description that the people or animals bitten by *D. acutus* could not walk more than 100 steps. More than 90% of the population of *D. acutus* is found in China, and the frequency of critical conditions and even death related to the bite of this snake is higher than that by many other venomous snakes [29]. AKI is the most serious systemic effect and common complication, which leads to secondary renal ischemia and failure. An enhanced knowledge of relevant information on AKI induced by *D. acutus* envenomation would contribute to the development of novel therapeutic approaches. However, in contrast to the considerable knowledge available on the nephrotoxicity of snake venom in general [30, 31], information on the AKI induced by *D. acutus* venom is rare, which led us to investigate this potential medicine that is still in the early stages of development.

In this study, we established an AKI model by intraperitoneally injecting *D. acutus* venom into mice to assess the complex and multifactorial pathogenesis of venom-induced AKI. Furthermore, the model provided a tool for investigating the protective effects of PCCC-CDs against AKI induced by *D. acutus* venom.

Current experiments have shown the development of substantial AKI with distinct changes in inflammatory cytokines and serum and urinary biochemical index, as well histopathological evidence of renal injury after intraperitoneal injection of snake venom [31]. These findings indicated the possible factors that may mainly contribute to the venom toxicity are [32] (1) direct venom cytotoxicity against the kidney and (2) renal inflammatory reactions.

Specifically, renal insufficiency was confirmed approximately 24 h post venom injection based on oliguria associated with proteinuria and elevated serum biomarkers (SCR and BUN). We further affirmed the renal involvement in the *D. acutus* venom-treated group using biochemical analysis, which showed significantly elevated UTP and MALB levels compared with those of the control group. These findings indicated the presence of glomerular malfunction and tubular reabsorption in the venom-treated group [33], which were supported by evidence of histopathological change. In contrast, a significant reduction in the levels of UTP and MALB was observed in the envenomated medium-dose PCCC-CD-treated group. In addition, serum biochemical indicators (SCR and BUN) are other vital parameters used to determine the elevation of renal dysfunction in AKI and they remain clinical indicators in its diagnosis [34]. Injecting snake venom from day 1 to 5 also dramatically increased the levels of SCR and BUN, whereas treatment with PCCC-CDs reversed these effects, resulting in a faster recovery than that of the control group. More importantly, the distinct changes in the kidney tissue, marked haemorrhage, renal tubular dilation, and degeneration further indicated the direct impairment of the kidney by the venom. The attenuating effects of PCCC-CDs on the histopathological changes were demonstrated in this study. These results suggested that PCCC-CDs inhibited the AKI-induced abnormal manifestation of urinary and serum biochemical markers associated with kidney dysfunction as well as renal histological damage. Furthermore, these effects may be attributable to the amelioration of the direct nephrotoxicity of the *D. acutus* venom by the PCCC-CDs [35]. The protective effects of PCCC-CDs were evidenced by the inhibition of *D. acutus* venom-induced direct kidney damage.

An intense inflammatory response is a common feature induced by envenomation by venomous animals such as snakes and caterpillars [35–37]. The signs of systemic inflammation with mononuclear cell infiltration, neutrophilic leukocytosis, tubular epithelial cell degeneration, and necrosis have also been shown in kidney impairment induced by injecting snake venom. MCP-1 is a small molecule protein that plays a vital role in recruiting and activating leukocytes during inflammatory responses [38]. In addition, mononuclear phagocytes and lymphocytes may contribute to acute renal cell injury by different mechanisms such as the secretion of proinflammatory mediators, which may then induce resident renal cells to express chemokines [39]. The involvement of MCP-1, inflammatory cytokines (IL-1β) and anti-inflammatory cytokine (IL-10) in the inflammatory response in the pathogenesis of AKI in mice injected with *D. acutus* venom only was demonstrated in the present study. This observation indicated that the underlying mechanism of the *D. acutus* venom-induced AKI may be associated with the renal inflammatory response. The evidence that exposure to PCCC-CDs significantly reduced levels of IL-1β, IL-10, and MCP-1 suggests that CDs may exhibit renoprotection by inhibiting renal inflammatory reactions.

Furthermore, PLTs play a crucial role in acute haemostatic and inflammatory processes and are associated with diverse inflammatory pathologies [40, 41]. They are highly sensitive and respond quickly to biological changes when an organism is injured or bleeding, as the first cells to arrive at the site of acute injury to interact with endothelial cells and leukocytes [42]. PLTs are involved in the pathogenesis of AKI [43], and are considered a prognostic marker that is significantly associated with a worse outcome of AKI [44]. This study provided evidence that *D. acutus* venom conspicuously decreased the PLT count, which was consistent with the results of studies reporting that thrombocytopenia can be induced by snake venom [34, 45]. We observed that exposure to PCCC-CDs significantly elevated the PLT counts, which was consistent with the findings of a previous study [23].

The abnormalities of AKI induced by *D. acutus* venom were, to our knowledge, demonstrated for the first time in the current study and mainly included renal dysfunction associated with proteinuria, oliguria, elevated BUN and SCR levels, pathological kidney damage, inflammatory responses, and thrombocytopenia.

Remarkably, the PCCC-CDs demonstrated protective activity against *D. acutus* venom-induced AKI by inhibiting the associated impairments, as evidenced in this study for the first time. This study was a preliminary evaluation of the beneficial effects of PCCC-CDs on AKI induced by *D. acutus* venom, and further investigations of the underlying mechanism would be the focus of future studies.

## Conclusion

The impressive protective effects of PCCC-CDs on *D. acutus* venom-induced AKI have been demonstrated in this study, for the first time, to the best of our knowledge. The AKI-related effects were mainly manifested as renal dysfunction, pathological kidney damage, inflammatory responses, and thrombocytopenia. These results indicated that PCCC-CDs have potential application prospects for use as a complementary medicine for the treatment of abnormalities induced by *D. acutus* venom-induced AKI. Furthermore, this provides a novel strategy for the study of active ingredients of traditional Chinese medicine formulations, and further broadens the biomedical applications of CDs.

## Data Availability

All data generated or analysed during this study are included in this published article.

## Abbreviations

AKI: Acute kidney injury
BUN: Blood urea nitrogen
CDs: Carbon dots
*D. acutus*: *Deinagkistrodon acutus*
HE: Haematoxylin and eosin
IL: Interleukin
MALB: Microalbuminuria
MCP-1: Monocyte chemotactic protein 1
PCC: Phellodendri Chinensis Cortex
PCCC-CDs: Phellodendri Chinensis Cortex Carbonisata-carbon dots
PLT: Platelet counts
SCR: Serum creatinine
UTP: Urinary total protein
